# Separating Macroecological Pattern and Process: Comparing Ecological, Economic, and Geological Systems

**DOI:** 10.1371/journal.pone.0112850

**Published:** 2014-11-10

**Authors:** Benjamin Blonder, Lindsey Sloat, Brian J. Enquist, Brian McGill

**Affiliations:** 1 Sky School, University of Arizona, Tucson, Arizona, United States of America; 2 Department of Ecology and Evolutionary Biology, University of Arizona, Tucson, Arizona, United States of America; 3 Santa Fe Institute, Santa Fe, New Mexico, United States of America; 4 School of Biology and Ecology, University of Maine, Orono, Maine, United States of America; Arizona State University, United States of America

## Abstract

Theories of biodiversity rest on several macroecological patterns describing the relationship between species abundance and diversity. A central problem is that all theories make similar predictions for these patterns despite disparate assumptions. A troubling implication is that these patterns may not reflect anything unique about organizational principles of biology or the functioning of ecological systems. To test this, we analyze five datasets from ecological, economic, and geological systems that describe the distribution of objects across categories in the United States. At the level of functional form (‘first-order effects’), these patterns are not unique to ecological systems, indicating they may reveal little about biological process. However, we show that mechanism can be better revealed in the scale-dependency of first-order patterns (‘second-order effects’). These results provide a roadmap for biodiversity theory to move beyond traditional patterns, and also suggest ways in which macroecological theory can constrain the dynamics of economic systems.

## Introduction

Decades of research have identified four central patterns that together describe the broad-scale organization of biological diversity [1]. These include the: abundance distribution of species [2]; the relationship between species richness and area [3], [4]; the decrease in assemblage similarity with increasing distance [5]; and the spatial dispersion of individuals within species [6], [7]. A central question is how local biotic and abiotic interactions and variation in rates of speciation and extinction influence these large-scale patterns of diversity [8]. Indeed, much of the ongoing debates within biodiversity science result from the fact that many different models have been proposed to explain these individual patterns [2], [4], [9]–[12]. More recently, several theories such as maximum entropy [13] and neutral theory [14]–[16] have claimed to be able to simultaneously predict these patterns [1].

A central problem for theories of biodiversity is that they all make similar predictions for these near-universal patterns despite beginning from disparate assumptions [17]. One potentially troubling implication for ecology is that these patterns may not reflect anything unique about organizational principles of biology or the functioning of ecological systems [11], [18], [19]. Instead, they may be a statistical inevitability for any complex system with a large number of variables influencing the system’s dynamics [20]–[22]. If non-ecological systems show similar patterns, then the fundamental validity of theories of biodiversity that invoke ecological mechanisms as an explanation would be challenged. Stronger tests of theory require alternative approaches.

There is an opportunity to identify a different set of patterns that arise from only ecological processes, and which can therefore distinguish between ecological and non-ecological systems [23], [24]. We hypothesize that distinguishing biodiversity theories using empirical patterns is possible with second-order effects but not with first-order effects. We define first-order effects as a set of functions that describe macroecological patterns across scales, and second-order effects as the scale-dependent parameters of these functions. We specifically hypothesize that:

(1) Any system where objects are partitioned in categories (species) across space and many variables interact multiplicatively will be described by a common set of functions, i.e. first-order effects. These first-order effects can be predicted based on common assumptions of multiple unified biodiversity theories [1] or are statistically inevitable consequences of the Central Limit Theorem [20], [21]. Thus, any system should be characterized by an approximate log-normal species-abundance distribution (SAD) [25], [26], an approximate [3], [27] power law [28] species-area relationship (SAR), a monotonically declining Jaccard similarity-distance function [5], and a positive intraspecific clustering function at different distances (i.e. clumped at all scales; see Methods) [6], [29]. Many biodiversity theories predict some or all of these patterns [1].
(2) Ecological and non-ecological patterns, however, can be separated by changes in these patterns change with scale. Thus, quantifying the scale-dependent parameters, i.e. second-order effects [7], [30]–[32] provide a novel way to assess mechanism in macroecology. For example, ecological processes (e.g. dispersal limitation, speciation) will have different scale dependences depending on the system of interest. Spatial scale may affect the slope of the species-area relationship [27] as well as the statistical moments of the species abundance distribution [33]. Some current unified theories of biodiversity are beginning to incorporate scale-dependence and these second-order effects into their predictions [13], [14] while for others the role of scale remains unclear [19], [34].

We hypothesize that ecological and non-ecological systems can be distinguished based on several patterns (Table 1). Our approach is to: 1) establish baseline expectations for first-order effects based on different biodiversity theories; 2) identify the potentially scale-dependent parameters of first-order effects; 3) plot these parameters as a function of spatial scale; and 4) detect changes in these functions from the baseline expectation. For example, the decay of similarity with distance pattern is predicted by several theories to be a negative exponential function (1). The slope of this function on logarithmic scale should be scale-invariant (2). However, a plot of the local slope of empirical data (3) might show a peak at large distance scales (4) indicative of a second-order effect that can only be explained by additional mechanisms not incorporated into the original biodiversity theories.

**Table 1:** First and second order effects.

*Macroecological pattern*	*First-order effect*	*Second-order effect*
Species abundance distribution	Log-normal (approximate)	Changes in mean, coefficient of variation, skewness, kurtosis at different area scales
Species area relationship	Power law (approximate)	Changes in local slope at different area scales
Similarity-distance relationship	Monotonic decreasing	Changes in local slope at different distance scales
Fraction of clumped species	Positive	Changes in local slope at different distance scales

First-order effects describe all datasets, while second-order effects may provide scale-dependent approaches for distinguishing datasets.

**Figure 1:**
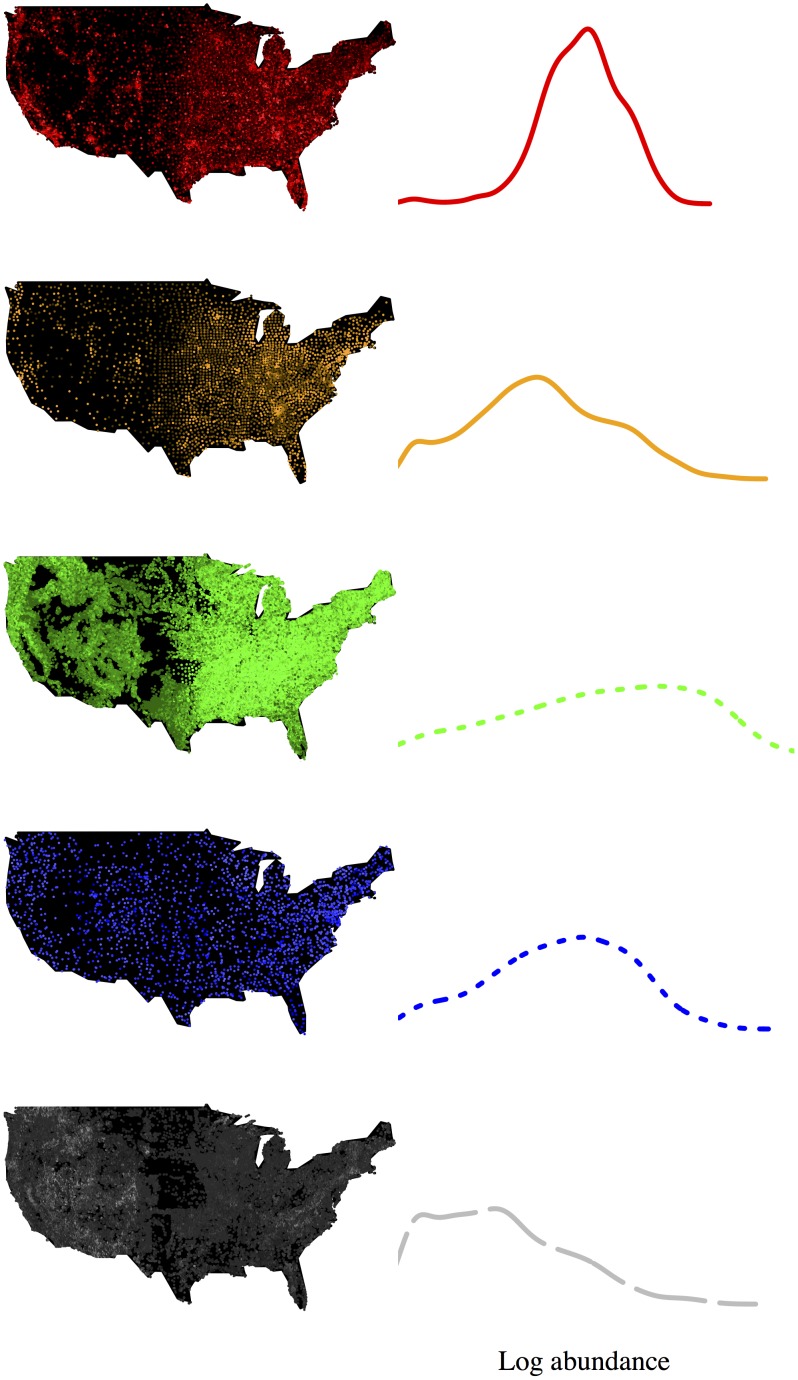
Distribution of objects across categories and space. Left column, site locations for each dataset (colored as described in Table 2). Site brightness is proportional to richness. Right column, relative abundance distribution for log-transformed abundance data at full scale (a first-order effect). All datasets are shown with the same axes.

Here we use this approach to provide general insights into the degree to which non-ecological systems can be explained by ecological theory. We compared first- and second-order effects across a broad set of ecological and non-ecological systems. We compiled five large datasets that each describe the abundance, location, and identity of objects in multiple categories (species) throughout the continental United States, encompassing two ecological systems (North American birds and trees), two economic systems (US Census county business patterns, and a commercial database of franchise locations for several hundred major corporations) and one geological system (USGS mineral resources database) (Table 2). These datasets were chosen because they are either complete censuses or are known to be well sampled, have very large number of objects and categories, occur over the same large region, and have high spatial resolution (Figure 1).

**Table 2:** Summary statistics for each economic, ecological, and geological dataset.

Dataset	Corporate locations	Industrial codes	Trees	Birds	Minerals
**Type**	Economy	Economy	Ecology	Ecology	Geology
**Lines drawn as**	solid red	solid orange	dotted green	dotted blue	dashed gray
**# Species**	455	3,777	384	584	746
**# Individuals**	660,935	7,628,863	11,887,262	1,640,449	587,571
**# Sites**	20,936	3,106	391,981	2,251	54,837
**Most common five species**	Subway, Shell,T-Mobile,McDonald’s,BP	Offices of physicians(exc mental health),Independent artists,writers& performers,Offices oflawyers,Offices ofdentists,Limited-servicerestaurants	Loblolly pine,Red maple,Sweetgum,Sugarmaple,White oak	Red-wingedblackbird,European starling,Americanrobin,Mourning dove,American crow	Gold, Sand & gravel,Construction, Silver,Copper

Maps display higher species richness at each site in brighter colors. In all subsequent graphs we have used the line-coloring scheme shown here.

## Materials and Methods

### Dataset assemblage

We generated community matrices for each of five datasets in which the *i*th row and *j*th column represented the abundance of species *j* at site *i*. Each community matrix was augmented with the latitude and longitude of each site. Each dataset was clipped to sites contained within the continental United States. All datasets were transformed from their raw form to community matrices using R for data manipulation and MATLAB for GIS analyses. These datasets are available in Information S1.

#### Dataset 1: Corporate locations

We purchased a commercial dataset containing the street addresses and latitude/longitudes of all locations of several hundred major corporations (AggData, Inc.). The data represent a census obtained between 2008 and 2010. We used shapefiles for the United States Census zip code tabulation areas (ZCTAs) to assign these point occurrences into assemblages. These approximately 20,000 areas cover the entire United States. While ZCTAs have unique complex boundaries and variable areas, they each cover roughly equivalent population levels and are a good comparable assemblage unit for this study. We then determined the latitude and longitude of each assemblage as the centroid of each ZCTA.

#### Dataset 2: Industrial codes

We downloaded the United States Census County Business Patterns dataset, which counts the numbers of businesses of different size classes in each of the North American Industrial Classification System’s (NAICS) nested categories, within each of the counties of every state of the United States. These data were valid for the 2007 census year. The data include some intentional inaccuracies (low-abundance data swapped between sites or abundances randomized) to comply with privacy laws, but these effects are small in magnitude and should not affect our analyses. We restricted our analysis to only the most specific (six-digit) level of NAICS classification in order to closely match between biological and business species. To further improve this correspondence, we also assumed that businesses that fell into different size classes (1–10, 10–100, 100–1000, 1000+ employees) within a given NAICS category represented different species. We also obtained shapefiles for county boundaries and determined the latitude and longitude of each assembly as the centroid of each county.

#### Dataset 3: Birds

We obtained data from the North American Breeding Bird Survey, which counts the abundance of the bird species observed along hundreds of multi-kilometer transect routes by multiple volunteer birders. We used data from the 2007 counts. We treated each route as an assemblage and determined its latitude and longitude as the midpoint of the route.

#### Dataset 4: Trees

We obtained data from the United States Forest Service’s Forest Inventory of America, which counts the abundances of several hundred species of trees at hundreds of thousands of plots across the United States. At each plot, we used data from its most recent census, which ranged from 1985–2008. Plot data were pre-corrected for variable plot size and only included live trees. Because of privacy laws, these data contain intentional inaccuracies (plots on private land have their coordinates fuzzed and their abundances swapped) that are small in magnitude and do not affect our analyses. We treated each plot as an assemblage and used the plot center for the assemblage latitude and longitude.

#### Dataset 5: Minerals

We downloaded data from the United States Geological Survey’s Mineral Resource Data System, which describes the locations of metallic and nonmetallic minerals throughout the world. We pooled the abundances of commodities, ores, and gangue at each site, because we were interested in geological processes and did not wish to stratify the data by economic value. Because each site contained a very low number of minerals (typically representing the useful output of a single mine) we chose to generate an equal-area grid (1000×1000) covering the bounding box of the continental United States, and pooled mineral abundances for all sites falling within each grid cell. We then defined the assemblage latitude and longitude as the center of the grid cell.

### Data analysis

#### Species-abundance distribution

We sampled the abundance distribution at 100 spatial scales that logarithmically spanned a range from 0.1° to 40°. At each scale, we chose 500 random sites. We defined a small circle on the surface of the earth whose radius was determined by the current spatial scale and whose center was the location of the current site. We then intersected this circle with a polygon defining the boundary of the region of interest (here, the continental United States). We calculated the surface area of this new polygon (in km^2^) using spherical geometry and the known radius of the earth to determine the effective area of the site. We then pooled abundances for all species at all sites enclosed within this polygon, applied a log transformation, and calculated the mean, coefficient of variation, skewness, and kurtosis of this distribution.

#### Species-area relationship

We determined the species-area relationship using an identical procedure as for the species-abundance distribution, but calculated species richness instead of abundance distribution moments within each polygon. We log-transformed both area and richness before analysis so that the local/global slope of the curve reflects the scale dependent/independent power-law scaling exponent.

#### Similarity-distance function

We sampled the similarity-distance function at 100 spatial scales that logarithmically spanned a range from 0.1° to 40°. At each scale, we chose 1000 random pairs of sites. We calculated the distance between assemblages (in km) as the minimum arc length along the surface of the earth joining the centers of these assemblages. Then, for each site within each pair of sites we generated small circles centered on the location of each site with a radius equal to the current spatial scale. We intersected each small circle with the boundary polygon of the region of interest (here, the continental United States). To obtain the assemblage area we used the sum of the areas of both polygons (in km^2^) calculated by the same approach described for the abundance distribution. We also pooled all abundances within each polygon and calculated the Jaccard similarity (number of species in common divided by the number of species in either polygon). To simplify the display of information results were plotted only for assemblages whose summed area was in the 10–100 km^2^ or the 1000 to 10000 km^2^ bins.

#### Intraspecific clumping

We assessed the fraction of species that exhibited intraspecific clumping at distances ranging from 10 to 5000 km in 10 km intervals. An individual species was defined to be intraspecifically clumped at a given distance scale if its observed pairwise distance distribution exceeded the upper 95% quantile of 100 samples from a null pairwise distance distribution. We calculated the pairwise distance distribution as the vector of distances (accounting for the curvature of the earth, as defined for the similarity-distance function) between every pair of sites at which this species occurred. We determined the null pairwise distance distribution by counting the number of sites at which this species occurred, randomly assigning that many occurrences of this species to randomly chosen sites, and repeating the pairwise distance calculation. This method accounts for sites that are non-uniformly or non-randomly positioned, corrects distances for the curvature of the earth, and generates conclusions that are consistent with more established methods for detecting clumping (e.g. pair correlation/o-ring function [6]). However, this method is computationally much faster for large datasets, because distance and intersection calculations can be pre-computed a single time.

#### First-order effects

Statistics were based on the metrics calculated using the methods described in the previous section. For the species-abundance distribution, we used abundance data at the largest spatial scale. We fit several candidate distributions to the data (Pareto, power-bend [35], Poisson log-normal, log-series, Weibull) and identified the distribution with the lowest AIC. For the species-area relationship, we used the log-transformed area and richness values described in the previous section, then reported the slope of the model (i.e. the power law exponent). For the similarity-distance relationship, we constructed a linear model using the sampled similarities and log-transformed distances for 10^4 ^km^2^ assemblages and reported the slope of the model. For these two analyses, all models were highly significant (p<0.05) but trivially so because of the very large degrees of freedom. For the clumping analysis, we reported the fraction of species that were significantly clumped at the 5000 km distance scale. Because of the very large number of degrees of freedom in all these analyses (up to 49,998), the standard error for every coefficient was much smaller than the coefficient value. We therefore did not present these uncertainty estimates or *p*-values because these statistical differences were unlikely to conclusively reflect biological differences.

#### Data visualization

We chose to show central trends in the data using LOESS (locally smoothed regression) and to quantify variation using error envelopes representing each middle quartile of a local subset of the data.

## Results

We first quantified first-order effects in each ecological, economic, and geological dataset neglecting the effect of spatial scale. Our analyses show that, when data are aggregating at a continental spatial scale, each dataset is characterized by the expected first order effects (see Methods for details). All species abundance distributions were best fit by a log-normal distribution (ΔAIC to the next-best distribution >30), except for the tree dataset for which a log-normal or Weibull distribution were both appropriate (ΔAIC = 4). All species-area relationships had log-log slopes ranging from 0.28 to 0.50. All distance decay relationships had log-linear slopes ranging from –0.17 to –0.72. Lastly, most datasets showed intraspecific clumping (7–52% of species significantly clumped). The only exception was for trees, but this dataset included many widely cultivated species. In contrast, we found that second-order effects can distinguish between these datasets, suggesting the operation of different processes structuring each system across a range of scales.

**Figure 2:**
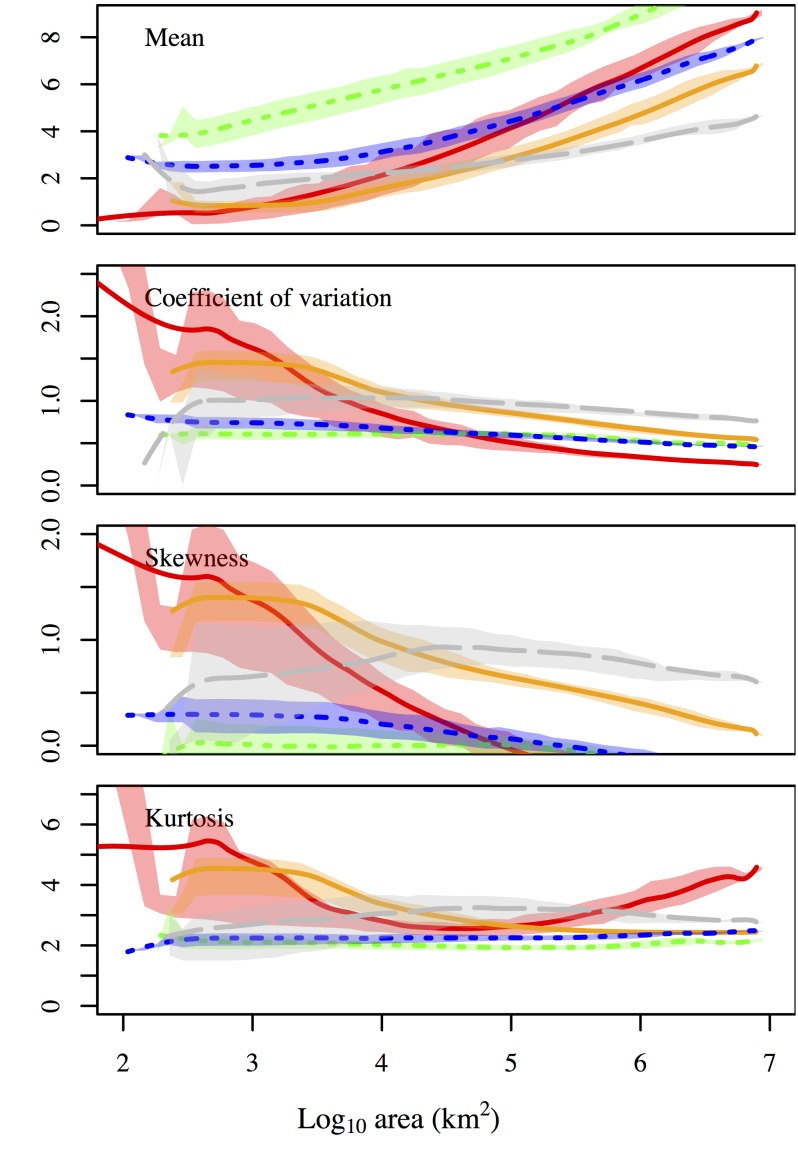
Central moments of the species-abundance distribution for log-transformed data. Line colors are described in Table 2.

We found that for scales of up to ∼10^5 ^km^2^ the coefficient of variation for the species-abundance distribution (Figure 2) for skewness, and kurtosis was larger for the economic datasets than for the ecological or geological datasets. These differences indicate the existence of a process specific to these economic systems that generates higher fractions of rare species at local scales. For example, it could be the case that rapidly growing economies have both higher birth and death rates reflected in large numbers of new businesses. However, all datasets had lower positive skewness at large scales, consistent with dispersal limiting the spread of rare species across space.

**Figure 3:**
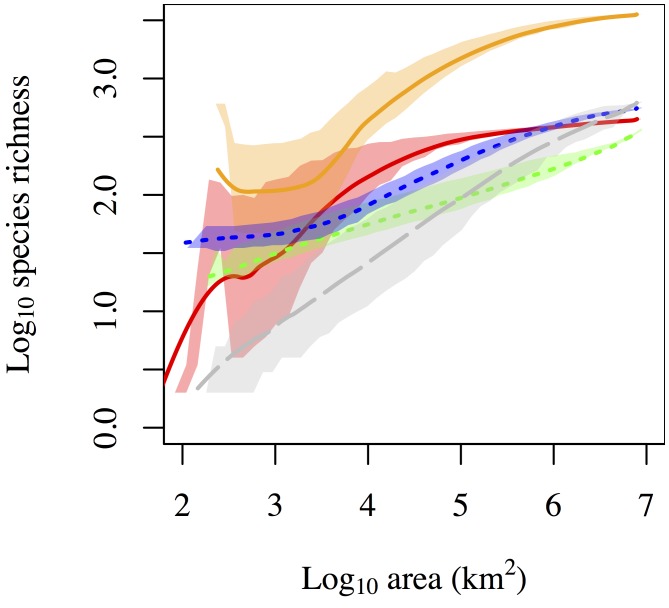
The species-area relationship distinguishes ecological datasets at large scales. Line colors are described in Table 2.

Across all systems, species-area relationships displayed a range of slopes and curvature indicating scale-dependent processes of richness accumulation (Figure 3). Consistent with dispersal limitation and a transition to novel species pools, we found a small increase in slope at intermediate scales for the biological data, A more striking pattern is the decrease in slope at large scales of the economic data, consistent with convergence to similar species pools on both coasts.

**Figure 4:**
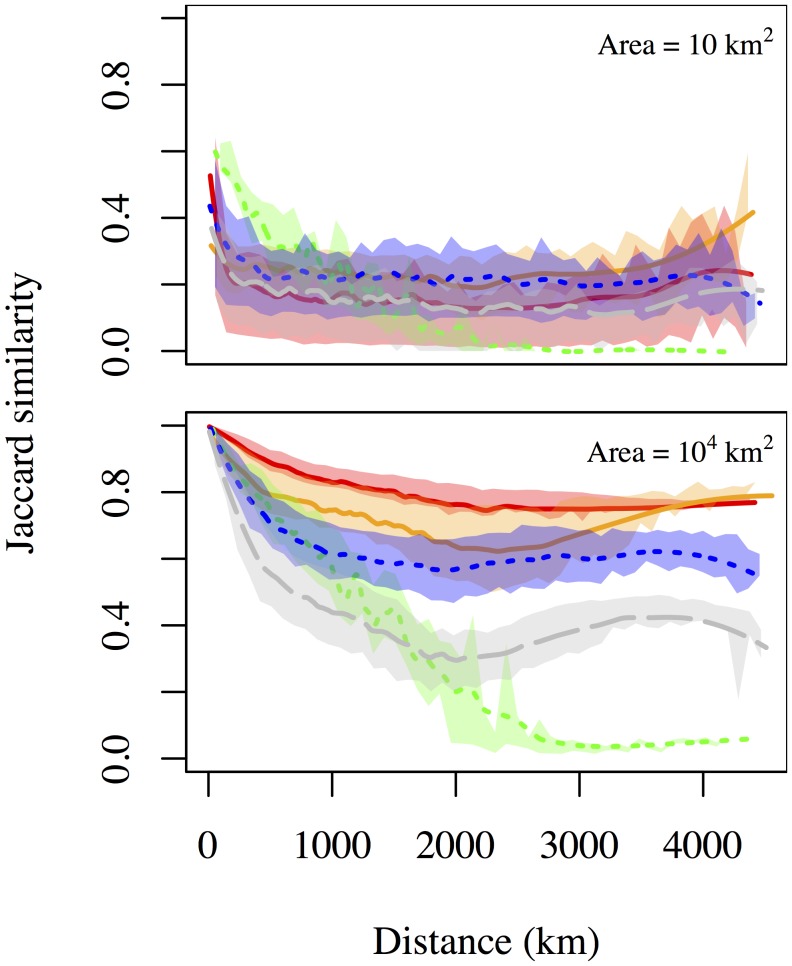
The decay in assemblage similarity with distance depends strongly on spatial scale. The rapidity of decrease and the minimum similarity are functions of dataset type and assemblage size. Line colors are described in Table 2.

The similarity-distance function showed a range of decay rates and minimum similarities when comparing datasets (Figure 4). Ecological datasets decayed faster than economic or geological datasets, and an ecological dataset (trees) had the lowest minimum similarity at large distances. Minimum bird similarity was higher than minimum tree similarity, presumably because of the high dispersal potential and large range size of many birds. Economic datasets showed an increase in similarity at very large distances, consistent with high similarity of species pools on both coasts. This change in similarity was weaker for the geological dataset. Changes in the similarity-distance function with assemblage area were also consistent with dispersal limitation being more important in biological systems. We found that in communities encompassing larger areas, ecological systems maintained their decay rates and reached comparable minimum similarities, but that economic systems exhibited very limited decay and high minimum similarity.

**Figure 5:**
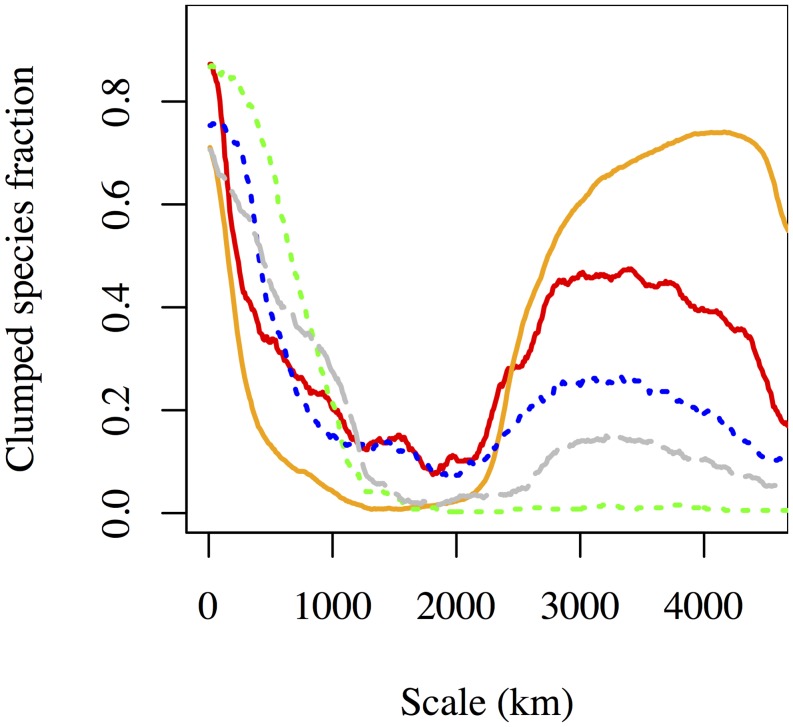
The fraction of species for which intra-specific clumping is consistently high at very small and very large scales. Among datasets, the clumping varies widely in magnitude with spatial scale. Line colors are described in Table 2.

The intraspecific clumping function showed more clumping at longer distances in ecological data sets compared to economic or geological datasets (Figure 5). The width of this leftmost part of the clumping function may provide insight into the average dispersal distance for species [6]. At intermediate distances, we found very low levels of clumping in all datasets, indicating that species distributions are spatially random at mid-continent scales. However, we also found increased clumping at whole-continent distances, especially in the economic datasets. This is broadly consistent with low dispersal limitation of businesses, and the high similarity of species pools on both coasts in economic systems.

## Discussion

We have shown that spatial scaling can successfully separate four universal macroecological patterns. The consistency of first-order effects across ecological, economic, and geological systems [23], [24] indicates that they provide little power to distinguish ecological mechanisms. We then identified second-order effects that were able to separate these five datasets – some of which are consistent with known ecological processes (e.g. dispersal limitation), and others not. Thus, future biodiversity theories should make simultaneous predictions for these scale-dependent second-order effects, in order to provide stronger tests and more separable predictions of theory. Our results indicate that theory especially should be able to make system-dependent predictions for the spatial scaling of in skewness and kurtosis of the species abundance distribution, as well as of the intraspecific clumping function. Spatial scaling can become a powerful approach to distinguish mechanisms and guide the development and testing of more complex theories of biodiversity.

The general lack of dispersal limitation evident in economic systems is consistent with an ‘everything is everywhere’ perspective on economic diversity. For example, more than 80% of the major corporations remain the same in pairs of county-sized (10^4 ^km^2^) communities at distances of up to 4500 km (Figure 4). Our results provide strong evidence for low beta diversity and high homogeneity of economic landscapes in the United States. Biodiversity theories will need to incorporate additional parameters to make scale-dependent predictions consistent with this finding.

Our results leave unresolved a potentially important zero-th order effect describing each system’s state variables: the number of species and individuals found in each dataset. Although the value of these numbers set the scale of all first-order effects they may also ultimately constrain levels of variation in second-order effects. However, in all major theories of biodiversity, the number of individuals and the number of species are treated as free parameters [1]. Addressing the origin of the zeroth-order effect may provide as much insight as addressing the origin of second-order effects [36].

Our results question the importance of the species concept is to macroecological theory. All biodiversity theories and macroecological patterns are expressed in terms of species and individuals. For ecological systems these are natural and potentially preferred scales for understanding a system. However, there are many possible ways to partition objects in to categories for non-ecological systems, and it is unclear if any particular aggregation method should be preferred. For example, individuals businesses can be aggregated into NAICS codes, but the taxonomy and resolution of these codes is necessarily a human choice. Thus, macroecological theory may be applied best to biological species. However, many biodiversity theories are derived from very limited or no biological processes, suggesting that they should apply equally well to any partitioning of objects in to categories (e.g. taxon-invariance in the species-area relationship [37], [38]). Therefore deviations from predictions, such as our second-order effects, should still reflect additional mechanisms.

We showed that our approach could be used for distinguishing different datasets or detecting situations where theory could be modified to better accommodate empirical data. We do not intend the approach to be used for null-hypothesis significance testing, i.e. statistically rejecting the null hypothesis of no second-order effects. There are two reasons: first, the specific form of first-order effects may depend on the exact mathematical formulation of a biodiversity theory which limits our ability to derive general equations; and second, the form of second-order effects is likely to be more interesting than simply rejecting the ecological null hypothesis. Nevertheless, it should be possible to develop a mathematical formalism to infer second-order effects, a goal that may be useful for the developers of next-generation biodiversity theories.

The similarities in economies and ecosystems may indicate a set of shared processes and constraints whose elucidation will have fundamental or practical implications [24]. Ecological principles and theories have been used to understand economic phenomena like competition, wealth distributions and the growth of cities [39]–[41]. Because many first-order effects seem to occur regardless of system, extant macroecological theory may have practical consequences for other economic systems, e.g. financial networks [42]. For example, current work on up-scaling inexpensive local measurements of biodiversity for conservation purposes [27], [43] may be relevant to economic reporting in developing regions, or in understanding the origin of other economic distributions [44]. Macroecological theory, because of its lack of intrinsic ecological mechanism, may also be applicable to many economic systems. In this way it may provide a more realistic understanding of limits to economic growth by identifying the first-order effects that provide universal and unavoidable constraints on economic systems, but also by identifying the zeroth- or second-order effects that may practically be modulated by policy shifts.

## Supporting Information

Information S1 Community matrices for each dataset and MATLAB code to replicate all analyses.
(ZIP)
